# The Effect of Delayed Surgical Debridement in the Management of Open Tibial Fractures: A Systematic Review and Meta-Analysis

**DOI:** 10.3390/diagnostics11061017

**Published:** 2021-06-02

**Authors:** Marios Nicolaides, Alexandros Vris, Nima Heidari, Peter Bates, Georgios Pafitanis

**Affiliations:** 1 Division of Orthopaedics, Barts and The London School of Medicine and Dentistry, Queen Mary University of London, London E1 2AD, UK; avris@nhs.net (A.V.); n.heidari@gmail.com (N.H.); peter.bates2@nhs.net (P.B.); 2 Department of Trauma and Orthopaedic Surgery, The Royal London Hospital, Barts Health NHS Trust, London E1 1FR, UK; 3 Group for Academic Plastic Surgery, Barts and The London School of Medicine and Dentistry, Queen Mary University of London, London E1 2AD, UK; g.pafitanis@qmul.ac.uk; 4 Department of Plastic Surgery and Reconstructive Surgery, The Royal London Hospital, Barts Health NHS Trust, London E1 1FR, UK

**Keywords:** open tibial fracture, surgical debridement, timing, open fractures, debridement, infection, non-union, orthoplastics, BOAST 4, lower limb trauma

## Abstract

Introduction: Open tibial fractures are complex injuries with variable outcomes that significantly impact patients’ lives. Surgical debridement is paramount in preventing detrimental complications such as infection and non-union; however, the exact timing of debridement remains a topic of great controversy. The aim of this study is to evaluate the association between timing of surgical debridement and outcomes such as infection and non-union in open tibial fractures. Materials and Methods: We performed a systematic review and meta-analysis of the literature to capture studies evaluating the association between timing of initial surgical debridement and infection or non-union, or other reported outcomes. We searched the MEDLINE, PubMed Central, EMBASE, SCOPUS, Cochrane Central and Web of Science electronic databases. Our methodology was guided by the Preferred Reporting Items for Systematic Reviews and Meta-Analyses statement and the Cochrane handbook for systematic reviews of interventions. Results: The systematic review included 20 studies with 10,032 open tibial fractures. The overall infection rate was 14.3% (314 out of 2193) and the overall non-union rate 14.2% (116 out of 817). We did not find any statistically significant association between delayed debridement and infection rate (OR = 0.87; 95% CI, 0.68 to 1.11; *p* = 0.23) or non-union rate (OR = 0.70; 95% CI, 0.42 to 1.15; *p* = 0.13). These findings did not change when we accounted for the effect of different time thresholds used for defining early and late debridement, nor with the Gustilo–Anderson classification or varying study characteristics. Conclusion: The findings of this meta-analysis support that delayed surgical debridement does not increase the infection or non-union rates in open tibial fracture injuries. Consequently, we propose that a reasonable delay in the initial debridement is acceptable to ensure that optimal management conditions are in place, such that the availability of surgical expertise, skilled staff and equipment are prioritised over getting to surgery rapidly. We recommend changing the standard guidance around timing for performing surgical debridement to ‘as soon as reasonably possible, once appropriate personnel and equipment are available; ideally within 24-h’.

## 1. Introduction

Open tibial fractures are complex injuries with variable outcomes that significantly impact patients’ lives. The subcutaneous position of the medial border of the tibia and the paucity of muscle attachments distally cause the bone to be vulnerable to becoming locally devascularised after high-energy trauma, which contributes to the increased rates of infection and non-union. Historically, it has been suggested that timely surgical debridement can reduce the various complications occurring after treatment [1]. Gustilo and Anderson stated that ‘adequate debridement is the single most important factor in the attainment of a good result in the treatment of an open fracture’ [1]. *Debridement* is derived from the French word débrider to ‘unbridle’; in surgical terms, ‘to release constrictions and tension in a wound by incision’ [2]. In modern surgery, it is better described as cleansing a wound by surgically excising dead or devitalised tissue, removing foreign material and irrigating to dilute contaminants. However, debridement has evolved to become much more than just wound cleansing. Surgeons are also mindful to minimise any additional detrimental effects of debridement that might unnecessarily threaten the viability of otherwise healthy tissue, such as preserving intact muscle, periosteum and perforator vessels. In addition, the ‘first debridement’ surgery has come to be recognised as an ideal moment for combined orthopaedic and plastic surgical decision-making, where plans and timeframes can be agreed between the specialists present.

Infection and non-union are two of the most common complications following management of open tibial fractures [3]. They significantly impact clinical outcomes, they burden the healthcare service financially and are associated with chronic pain, opiate and alcohol misuse and subsequent unemployment and psychological problems [4,5]. Surgical debridement, along with antibiotic prophylaxis, are paramount in preventing these catastrophic complications [6]. The latest standards suggested administering intravenous antibiotics within 1 h following injury, rather than within 3 h as originally proposed [7]. This change has been driven by evidence demonstrating that early antibiotic prophylaxis significantly reduces post-operative infection in patients with open fractures of the extremities [8,9]. However, as per surgical debridement, the exact timing of debridement remains a topic of great controversy.

Until recently in the UK (2009 BOAST-4 guidelines), surgical debridement was preferentially performed within 6 h following injury. This threshold was largely based on an 1898 experimental study on guinea pigs, which demonstrated that open fracture wounds reach infection threshold at about 6 h after the injury due to the incubation period of bacteria [10]. The implementation of this ‘6-h rule’ was challenged by new evidence and geographical constraints around resources [11], which led to sequential revisions of the guidelines. In 2009, the UK open tibial fracture guidance recommended immediate surgical debridement for highly contaminated and vascular-compromised wounds, but debridement within 24-h for other open fractures; a move away from the 6-h mandate [12]. In 2017, the guidance was changed again, this time advocating debridement within 12-h for high-energy injuries (which would include the majority of open tibia fractures); and within 24-h for low-energy open fractures [13]. This change was also associated with a paradigm shift from rapid emergency surgery, to urgent transfer to an Orthoplastic service to facilitate a stepwise and disciplined management approach [14]. There was a move away from the traditional emphasis of getting to surgery quickly, instead prioritising expertise, Orthoplastic collaboration and performing debridement to a high level of proficiency.

Surgeons seem to agree on performing debridement at a reasonable time following injury; however, unnecessarily narrow time frames can hinder optimal management conditions, such as allowing appropriate staff/personnel to be present. Previous meta-analyses were inconclusive on the effect of delayed surgical debridement and surgical outcomes in lower-extremity open fractures [15,16,17]; however, since their publication, new evidence has emerged. The aim of this study is to evaluate the association between timing of surgical debridement and outcomes such as infection and fracture non-union, or any other reported surgical or functional outcome.

## 2. Materials and Methods

### 2.1. Study Design

We performed a systematic review and meta-analysis of the literature in accordance with the Preferred Reporting Items for Systematic Reviews and Meta-Analyses (PRISMA) Statement of 2009 [18] and the Cochrane Handbook for Systematic Reviews of Interventions [19]. Our work was guided by a prospectively developed protocol, registered in the PROSPERO database (CRD 42020191104).

### 2.2. Inclusion Criteria

We aimed to capture randomised or quasi-randomised controlled trials, cohort or case-control studies and case-series, evaluating the association between timing of initial surgical debridement and infection or non-union, or other reported outcomes, in the management of open tibial fractures. Studies were included if they reported: (a) Outcome frequency by time to surgical debridement following injury (i.e., infection rate in group receiving debridement in <6 h following injury versus >6 h), (b) mean/median time to surgical debridement in a group with positive outcome versus one without positive outcome and (c) any association between timing of surgical debridement and the event of infection or non-union presented in odds ratios or other measurable means. Studies were only reviewed if the published manuscript was in English. We excluded any studies that did not meet the above criteria and studies on animals. Furthermore, we excluded studies that recruited solely participants of less than 16 years of age and studies with less than 25 fractures.

### 2.3. Study Identification and Selection

We performed a comprehensive search of the literature on 13 May 2020. We searched the MEDLINE (via Ovid), PubMed Central, EMBASE, SCOPUS, Cochrane Central Register of Controlled Trials (CENTRAL) and Web of Science Core Collection electronic databases. Our search strategy included a combination of keyword terms and Medical Subject Headings (MeSH), such as open fractures, tibia and debridement (Supplementary Tables S1–S6). Our database search was supplemented by screening the bibliographies of previous systematic reviews and of published studies for relevant titles, searching clinical trial registries for ongoing trials (Clinical Trials Gov., ISRCTN, EU Clinical Trial Register) and a random search on Google Scholar.

Captured studies from our electronic database and manual search were exported and merged into a reference manager library (EndNote X9, Clarivate Analytics, Philadelphia, PA, USA). Duplicates were removed and screened by three independent reviewers at two levels: Title–abstract and full-text screening. Any discrepancies during title–abstract screening stage were resolved by including the article by default, whereas during full-text screening, they were resolved by discussion and senior author consensus. When both screening stages were completed, we searched for any relevant retraction statements, errata and duplicate reports for all included studies.

### 2.4. Data Collection

All relevant data were extracted using piloted forms and exported to a digital spreadsheet (Microsoft^®^ Excel). Data extraction was performed by two independent reviewers. We classified extraction fields into four main categories: Study characteristics and methods, population demographics, debridement and surgical intervention, and outcomes and results. Any discrepancies in the extracted data were resolved by thoroughly inspecting the manuscripts during reviewer meetings. Several studies did not provide data for tibias only, either because they looked at all lower-extremity fractures or at all open fractures overall; thus, in such cases, data extraction was deemed unfeasible. For these studies, we attempted to collect the relevant data by (a) looking at previous systematic reviews and (b) contacting the corresponding author, or any other author available, via email or through a social networking site for scientists and researchers (Research Gate). If no response was received, the study was excluded.

### 2.5. Risk of Bias Assessment and Quality of Evidence

Our review did not capture any randomised controlled trials (RCTs), thus the ROBINS-I tool for assessing risk of bias in non-randomised studies was used [20]. We stratified the risk in a traffic light configuration for confounding, selection of participants into the study, classification of interventions, deviations from intended interventions, missing data, measurement of outcomes, selection of the reported result and overall bias. Overall risk of bias was considered low risk if all domains were determined as low risk; moderate risk if at least one of the domains was determined as moderate risk but none as serious; serious risk if at least one of the domains was determined as serious risk but none as critical; and critical risk if at least one of the domains was determined as critical risk. The Grading of Recommendations Assessment, Development and Evaluation (GRADE) tool was used to rate the quality of evidence and produce a level of certainty for each outcome [21].

### 2.6. Data Synthesis and Analysis

We summarised the characteristics of each study in the PICO format and described them using descriptive statistics. Subsequently, we synthesised data quantitatively for primary outcomes (infection and non-union) and qualitatively for secondary outcomes (amputation, flap failure, length of hospital stay). A meta-analysis using a DerSimonian and Laird random effects model was performed to compare infection and non-union rates between groups that received early or late initial surgical debridement (i.e., group receiving debridement <6 h following injury versus >6 h) and to compare mean debridement times between groups positive and negative to the assessed outcome. A Hartung–Knapp adjustment for random effects model was applied to produce more adequate error rates in a small number of studies analysis [22]. Furthermore, a continuity correction of 0.5 was applied in studies with zero cell frequencies. If no events were reported in either group, then the study was not included in the meta-analysis, as such studies do not provide any indication of either the direction or magnitude of the relative treatment effect [19]. Study, subgroup and summative odds ratios (OR) and 95% confidence intervals (CI) were calculated and reported. All means were reported along with their standard deviation (SD).

In regard to overall infection, a subgroup analysis was performed for debridement time thresholds of 5, 6, 8, 12 and 24 h, calculating the mean effect for each group and comparing it across each other using the Q-test. For studies reporting multiple thresholds, we used the 12-h threshold where available—this was based on the latest BOA/BAPRAS guidance recommending initial surgical debridement in less than 12 h for high-energy open fractures [7]. Subgroup analyses were also performed for prospective and retrospective studies, those performed before and after 2010, and studies of different geographical location. Additionally, sensitivity analyses were performed for all time thresholds independently, studies reporting the use of cultures to confirm infection, studies that specified that they measured deep infection only, studies that specified the use of an Orthoplastic approach and studies that did not perform any debridement or irrigation on patients prior to entering the operating theatre. 

In regard to overall non-union, subgroup analyses were performed for debridement time thresholds of 6 and 8 h; prospective and retrospective studies; studies performed before and after 2010; and of different geographical location. Additionally, sensitivity analyses were performed for studies that specified the use of an Orthoplastic approach and for studies that did not perform any debridement or irrigation on patients prior to entering the operating theatre.

We assessed heterogeneity using the I^2^ statistic and Cochran’s Q test. For I2 values, we defined 0% to indicate no heterogeneity, 25% low, 50% moderate and 75% high [23]. Forest plots were plotted to qualitatively assess heterogeneity and to provide summary estimates. Publication bias was assessed visually by producing contour-enhanced funnel plots [24] and statistically using the Egger’s regression test for asymmetry [25]. We looked for statistical outliers by looking at the studies’ confidence intervals—we considered a study a statistical outlier when its confidence interval did not overlap with the 95% confidence interval of the pooled effect. Furthermore, we performed influence analyses tests to identify studies that exert a very high influence on our overall results. We have set the significance level for all above statistical tests to be 0.05. We used the R software for all statistical tests (Version 4.0.2 for Mac, The R Foundation for Statistical Computing).

## 3. Results

### 3.1. Search

**Figure 1:**
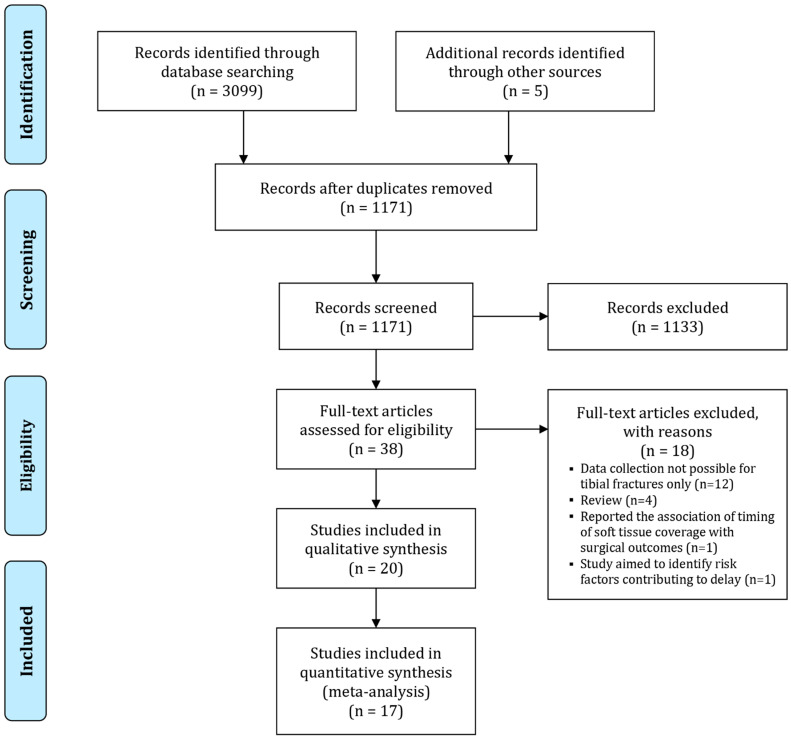
PRISMA flow diagram of study selection.

Our initial database search yielded a total of 3099 records, which were supplemented with 5 records from the manual search (Figure 1). Following duplicate removal, 1171 records remained. These were screened in their title and abstract against pre-defined eligibility criteria. We retrieved the full texts of 38 records to screen in their entity, whereas 1133 records were excluded. Out of the 38 articles, only 20 were deemed appropriate for inclusion. We excluded a total of 18 studies. The most common reason for exclusion was studies reporting data on all bones rather than tibial fractures only (*n* = 12) (Figure 1).

### 3.2. Characteristics of Included Studies

The total number of reported open tibial fractures in all studies was 10,032, ranging from 41 to 7560. Key characteristics of the 20 included studies are summarised in Table 1. Nineteen out of the 20 studies reported outcomes for infection, out of which 11 narrowed it down to deep infection. Eight studies reported non-union, one reported flap failure, one secondary amputation and one length of stay. Sixteen studies reported the Gustilo Anderson grading distribution of tibial fractures, however, only eight took it into consideration when analysing their data. In total, there were 253 Type I, 380 Type II, and 819 Type III (IIIA = 253, IIIB = 380, IIIC = 32, Unspecified = 154) reported tibial fractures.

Of the 20 included studies, only 5 provided a description of the debridement carried out [26,27,28,29,30]. Only three studies specified that an Orthoplastic approach was used in the management of fractures [31,32,33]. Six studies did not specify the method of skeletal fixation [32,34,35,36,37,38], whereas the rest used various methods for the management of the reported fractures. Furthermore, only two studies specified the method of soft tissue reconstruction [28,31]. All but one study [34] specified the use of antibiotic prophylaxis.

### 3.3. Risk of Bias Assessment

Risk of bias assessment was performed using the ROBINS-I tool for non-randomised controlled trials. Most studies were judged to carry serious bias (*n* = 13), while the remaining seven studies moderate (Supplementary Figure S1). We did not judge any study of possessing low or critical bias. The overall bias was determined by the ‘confounding’ and ‘deviation from intended interventions’ domains in all cases.

### 3.4. Infection

Infection was assessed by 19 studies out of these, 17 reported the frequency of infection in groups, which received early and late debridement. These reported a total of 2193 open fractures, 1376 debrided early and 817 late. Different time thresholds were used for differentiating between early and late debridement (Table 1). Only three studies [28,29,39] assessed infection using microbiological samples; the rest diagnosed infection by clinical signs. 

**Figure 2:**
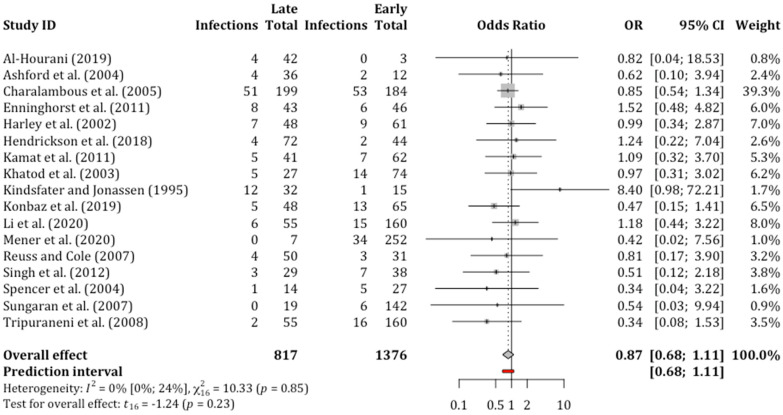
Forest plot of random effects meta-analysis comparing the incidence of developing infection after late surgical debridement compared to early debridement in open tibial fractures. OR = odds ratio; CI = confidence interval.

We did not find any association between delayed debridement and infection rate. Overall, 314 out of 2193 (14.3%) open tibial fractures got infected. The infection rate was marginally higher in the late debridement group (121 out of 817, 14.8%) compared to the early debridement group (193 out of 1376, 14.0%) when all 17 studies were included in the analysis. However, this difference was not statistically significant as per the relative mean effect (OR = 0.87; 95% CI, 0.68 to 1.11; *p* = 0.23) (Figure 2) or anticipated absolute effect (Table 2).

Two studies reported mean time to debridement in groups with infection compared to groups without infection [31,35]. The overall effect was not statistically significant (standardised mean difference = −0.0135; 95% CI, −0.1065 to 0.0795; *p* = 0.32). No heterogeneity was observed on the forest plot or on performing the Cochran’s Q test and I^2^ statistic (Q = 0; *p* = 0.9766; I^2^ = 0%). No publication bias was observed on inspection of a funnel plot.

Bednar and Parikh [27] and Duyos et al. [40] did not provide raw data on the number of tibial fractures infected or debrided within each time group and, thus, were not included in the meta-analysis. However, they reported no statistically significant difference in infection rate between early and late debridement groups.

### 3.5. Non-Union

Non-union was assessed by frequency in eight studies; however, one study [31] did not report non-union events in either debridement group and was, as per our methodology, excluded from the statistical analysis. The remaining seven studies reported a total of 817 open fractures, 399 debrided early and 418 late. Included studies used different time thresholds for differentiating between early and late debridement (Table 1). Five out of eight studies that reported non-union, specified the use of radiography for diagnosis [31,35,41,42,43].

**Figure 3:**
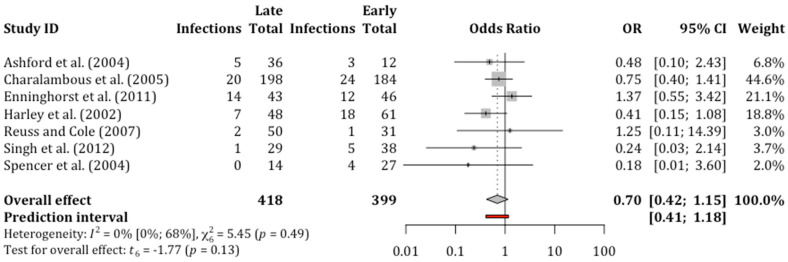
Forest plot of random effects meta-analysis comparing the incidence of non-union after late surgical debridement compared to early debridement in open tibial fractures. OR = odds ratio; CI = confidence interval.

We did not find any association between delayed debridement and non-union. An overall non-union rate of 14.2% (116 out of 817) was noted across all studies. The non-union rate was higher in the early debridement group (67 out of 399, 16.8%) compared to the late debridement group (49 out of 418, 11.7%). However, this difference was not statistically significant as per the relative mean effect (OR = 0.70; 95% CI, 0.42 to 1.15; *p* = 0.13) (Figure 3) or anticipated absolute effect (Table 2).

Only one study assessed the mean time to debridement in groups with non-union compared to groups with typical union, reporting no statistically significant outcomes (*p* = 0.08) [35].

### 3.6. Subgroup and Sensitivity Analyses

We performed several subgroup and sensitivity analyses. No statistically significant differences were noted when we looked at the effect of different time thresholds used for defining early and late debridement, the severity of fracture using the Gustilo–Anderson classification or the varying study characteristics; such as study design, publication year and country, use of cultures to confirm infection, use of an Orthoplastic approach, studies that measured deep infection only and studies that did not perform any debridement or irrigation on patients prior to entering the operating theatre.

### 3.7. Heterogeneity, Outliers and Publication Bias

No heterogeneity was observed on the forest plot or on performing the Cochran’s Q test and I^2^ statistic (Infection: Q = 10.33, *p* = 0.85, I^2^ = 0%; Non-union: Q = 5.45; *p* = 0.49; I^2^ = 0%). No extreme effect sizes (outliers) were detected as all included studies’ 95% confidence intervals overlap with the 95% confidence interval of the pooled effect. No publication bias was observed on inspection of a funnel plot or upon performing the Egger’s regression test for asymmetry (Infection: *p* = 0.85; Non-union: *p* = 0.34).

### 3.8. Secondary Outcomes

Only one study assessed the effect of delayed surgical debridement on amputation. [34] Following adjusted analyses for patient and hospital characteristics and clinical risk factors, they reported that timing of the first surgical debridement after 24 h is associated with more than three times greater odds of amputation (OR = 3.81; 95% CI, 1.80 to 8.07; *p* < 0.001) compared to patients having initial procedures before 24 h.

Al Hourani et al. reported that the time to initial debridement was lower in those who developed infection-associated flap failure, 15.8 h (SD = 8.01), compared to those who did not, 19.0 h (SD = 12.6) (*p* = 0.724, not statistically significant). However, they reported a statistically significant increase in time to initial debridement in one case of vascular-associated flap failure (*p* = 0.007).

Ashford et al. reported a longer length of stay in hospital, 51 days (range, 8 to 198) in the group debrided in less than 6 h, compared to the group debrided in more than 6 h, 49 (range, 3 to 171); not statistically significant.

## 4. Discussion

### 4.1. Main Findings

We performed a systematic review and meta-analysis of 10,032 open tibial fractures to evaluate the association between the timing of initial surgical debridement and infection, non-union or other reported outcomes. Our findings support that delayed surgical debridement results in little to no difference in infection or non-union rates at various time thresholds of up to 24 h (Table 2). We acknowledge that all included studies were cohort studies with variation in population demographics, intervention and outcome assessment. In an attempt to mitigate a number of these biases, we performed several subgroup and sensitivity analyses to demonstrate that our findings are not dependent on arbitrary or unclear decisions, or varying characteristics of the included studies. We trust that the subgroup and sensitivity analyses performed were focused and accomplished their purpose; yet the limited standardisation in the methodology of included studies inevitably lowers the quality of the evidence, which we judged to be very low (Table 2). The secondary aim of this study was to evaluate the association between the timing of initial surgical debridement in open tibial fractures and any other reported outcomes, including amputation, length of hospital stay and flap failure. Unfortunately, we did not reach a consensus for these outcomes as they were represented by one study each.

### 4.2. Findings of Excluded Studies

A recent national cohort study of 661 Gustilo-Anderson Type IIIB and IIIC open tibial fractures managed at major trauma centres of the UK, demonstrated a mean time to debridement of 20.93 h (SD = 41.78, Median = 12.12 h, IQR = 5.39–20.92) following injury [44]. Although they were not eligible for inclusion in our meta-analysis, they reported that the rate of infection was not statistical significantly different in fractures debrided within 12-h compared to after 12 h. Furthermore, out of the 12 studies we excluded for not providing data for tibial fractures, 10 (83%) reported no statistically significant associations between a delay in debridement and infectious or non-union complications [45,46,47,48,49,50,51,52,53,54]. Although these results are for all open or lower extremity fractures, they are in alignment with our findings for tibial fractures.

### 4.3. Comparison with Previous Meta-Analyses

Our findings also come in line with previously published meta-analyses. Prodromidis and Charalambous reported no statistically significant differences between 184 open tibial fractures debrided early (<6 h) compared to 199 open tibial fractures debrided late (>6 h), with regards to overall infection rate (risk ratio = 1.32; 95% CI, 0.54–3.23; *p* = 0.55), deep infection rate (risk ratio = 0.99; 95% CI, 0.48–2.07; *p* = 0.98), and non-union rate (risk ratio = 1.49; 95% CI, 0.64–3.49; *p* = 0.36) [17]. A previous meta-analysis by Shenker et al. reported similar findings for all open fractures overall and for tibial fractures only [16]. As in our review, their included studies used various thresholds to differentiate between early and late infection. They reported that the weighted cumulative odds ratio of infection after late debridement for tibial fractures was 0.89 (95% CI, 0.5 to 1.57), whereas for all open fractures overall was 0.91 (95% CI, 0.70 to 1.18)—both were not statistically significant [16].

### 4.4. Strengths and Limitations of This Study

To the best of our knowledge, this systematic review and meta-analysis is the largest to date to look at the effect of late surgical debridement on post-operative infection and non-union. Although the large number of cases was accompanied by extensive variability, we validated the overall effect by performing subgroup and sensitivity analyses, demonstrating that any arbitrary or unclear decisions or varying characteristics of the included studies were not significant to influence our findings. We have summarised new evidence concluding that delayed debridement does not lead to increased rates of infection or non-union. Our findings raise questions for the current UK recommendations for immediate surgical debridement.

The main limitations of our meta-analysis lie with those of the included studies. All studies were prospective and retrospective cohort studies, and not randomised controlled trials. We found variation in the demographics of the recruited population, intervention applied and method of assessing infection and non-union, concluding that all studies demonstrated moderate to serious risk of bias. Furthermore, our subgroup and sensitivity analyses did not justify the method or timing of skeletal stabilisation and soft tissue reconstruction used, the varying patient demographics and comorbidities or the various fracture characteristics (tibial location and mechanism of injury). Finally, the 95% confidence interval of both the relative and absolute effects of our meta-analysis includes the probability of no effect (i.e., OR = 0). A combination of the above factors deems our results to be of very low certainty based on the GRADE quality of evidence assessment. Nevertheless, taking into consideration the existing evidence in the literature on this topic, we are confident that no other review can yield a higher certainty in their reported results.

### 4.5. Interpretation of Results and Current Evidence

Even though we should take the findings of this review with caution—considering that the level of certainty is very low to the best of our knowledge this is the most up-to-date and comprehensive evidence on the effect of delayed debridement in the management of open tibial fractures. We have demonstrated that a delay in surgical debridement at various time thresholds of up to 24 h results in little to no difference in infection and non-union rates. One would argue that these results are peculiar, as there is evidence that an open wound colonised with bacteria gets progressively contaminated, as bacteria grow exponentially and increase the probability of infection [55,56]. Attempting to explain that a delay in surgical debridement can have beneficial effects would seem unjustified and irrational. This leaves two possible explanations: (a) There are other factors associated with infection and non-union, which mask any actual benefits of early debridement, or (b) timing is indeed of minor importance in relation to the quality of debridement.

It is difficult to argue for the former, as several of the included studies ruled out variables such as age, sex, laterality, multiple fractures, smoking, fracture location, type of flap, type of skeletal stabilisation and method of tissue reconstruction [30,31,35,41,42]. However, noteworthily, only four of the included studies adjusted for time to definitive soft tissue cover [29,31,32,36], an important factor thought to contribute to infection. Thereafter, it was not possible to adjust for it in our analysis and adds to the limitations of our findings.

The first to argue for the second point was Merrit in 1988, while attempting to identify factors that increase the risk of infection in patients with open fractures [57]. Interestingly, they found that the infection rate is related to the number of bacteria after wound debridement, rather than the number of bacteria before debridement [57]. The ‘timing versus quality’ balance might indeed play a bigger role than just timing. The quality of debridement is subjective to each surgeon, particularly as per its aggressiveness. We did not find any recent study in the literature assessing the association between the quality of surgical debridement, or the team performing it, and surgical outcomes. Furthermore, one study found that open fracture infections are mostly nosocomial in origin and the wound bacteria change while the patient is hospitalised [58]. Taking the above into consideration, we can argue that we should shift our efforts towards how well the patient is managed after being hospitalised, rather than the length of time from injury to the operating theatre [30].

### 4.6. Implications of Our Review

There are circumstances in which early surgical debridement might not be optimal or even possible [16]. Injuries taking place in rural settings or during military conflict might not have the required resources to manage open tibial fractures appropriately [59,60,61]. The transfer of these patients to major Orthoplastic centres seems likely to be beneficial for the patient, even at the expense of a delay in surgical debridement. In an ideal scenario, both orthopaedic and plastic surgery teams would work in unison at all patient management stages: Preoperative planning, intraoperative decision-making and post-operative care and follow-up [62]. This approach in limb salvage can improve outcomes such as pain, function and reduce length of hospital stay, post-operative complications and secondary procedures [63,64,65,66,67].

Furthermore, many patients present with open fractures outside normal working hours. Operating after-hours has well-described negative effects, including next-day fatigue of the personnel, increased workload for decision-making and scheduling, limited staff and equipment, increased surgical complications and mortality as well as economic implications [68,69,70,71,72]. Operating on these patients the next day can be justified, in view of our findings herein.

### 4.7. Impact on National Recommendations

Current UK recommendations suggest immediate debridement for highly contaminated open fractures, within 12 h for high-energy and within 24 h for low-energy open fractures. Even though the inherent limitations of the included studies in our review hinder us from invalidating these set-hour rules conclusively, our findings provide grounding for future reconsideration and revisions. We continue supporting urgent surgical care for all open tibial fractures; however, we find that a reasonable delay in the initial debridement is acceptable to ensure optimal management conditions. In light of our findings, we propose that a reasonable delay in the initial debridement is acceptable to ensure that optimal management conditions are in place, such that the availability of surgical expertise, skilled staff and equipment are prioritised immediacy of surgical intervention. We recommend changing the standard guidance around timing for performing surgical debridement to ‘as soon as reasonably possible, once appropriate personnel and equipment are available; ideally within 24-h.’

### 4.8. Future Research and Direction

Our findings have raised several questions around the co-existing factors that contribute to infection and non-union in open tibial fracture injuries. We speculate that factors such as time to definitive soft tissue coverage might have masked any actual benefits of early debridement in our data. Future research should focus on identifying these factors in large, exceptionally designed, prospective trials. We acknowledge that an RCT comparing early versus delayed debridement would be impractical to run with complex ethical implications. However, we encourage future research to continue evaluating the association between timing of surgical debridement and infectious or non-union complications, by standardising for factors such as population demographics, patient comorbidities, technical aspects of surgical debridement and outcome assessment. We also support that any future findings should be reported in correlation to the initial injury characteristics. Finally, we encourage future trials to assess the effect of varying technical factors of debridement (e.g., aggressiveness of soft tissue excision, surgical team members present and timing of definitive soft tissue cover) on surgical outcomes, as we identify a lack of these studies and trust that these factors might play a significant role.

## 5. Conclusions

The findings of this systematic review and meta-analysis support the proposal that a delay in surgical debridement does not increase the infection or non-union rate in open tibial fractures. On the basis of this meta-analysis, the current UK recommendations for urgent debridement of high-energy fractures have little support in the existing literature; however, the inherent limitations of the included studies hinder us from invalidating these set-hour rules conclusively. We continue supporting urgent surgical care for all open tibial fractures, but we propose that a reasonable delay in the initial debridement is acceptable to ensure that optimal management conditions are in place, such that the availability of surgical expertise, skilled staff and equipment are prioritised over getting to surgery rapidly. We recommend changing the standard guidance around timing for performing surgical debridement to ‘as soon as reasonably possible, once appropriate personnel and equipment are available; ideally within 24-h’.

## Figures and Tables

**Table 1:** Summary of characteristics of included studies.

Author	Year	Country	Study Design	Fractures	Gustilo Anderson					Time Threshold	Outcomes
					I	II	IIIA	IIIB	IIIC		
Al-Hourani et al. [29]	2019	UK	Retrospective	45	0	0	0	45	0	6	Deep infection Non-union Flap failure
Ashford et al. [24]	2004	Australia	Retrospective	48	3	10	14	21	0	6	Infection Non-union/delayed union Length of stay
Bednar and Parikh [25]	1993	Canada	Retrospective	52	NR					6	Deep infection
Charalambous et al. [37]	2005	UK	Retrospective	383	33	38	64	0	0	6	Infection Deep infection Secondary procedure to promote bone union
David Sears et al. [32]	2012	US	Retrospective	7560	NR					24, 48, 96 and 120	Amputation
Duyos et al. [38]	2017	Puerto Rico	Retrospective	227	NR					48, 72 and 96	Deep infection
Enninghorst et al. [33]	2011	Australia	Prospective	89	21	27	18	21	1	6	Deep infection Non-union
Harley et al. [39]	2002	Canada	Retrospective	109	19	53	37			8	Deep infection Non union
Hendrickson et al. [30]	2018	UK	Retrospective	116	0	0	0	116	0	12	Deep Infection
Kamat et al. [40]	2011	New Zealand	Retrospective	103	49	32	22			6	Infection
Khatod et al. [26]	2003	US	Retrospective	101	17	46	23	8	7	6	Infection
Kindsfater and Jonassen [27]	1995	US	Retrospective	47	0	25	13	7	2	5	Deep infection (osteomyelitis)
Konbaz et al. [41]	2019	Saudi Arabia	Retrospective	113	13	45	20	28	7	6	Infection
Li et al. [28]	2020	China	Retrospective	215	62	98	26	25	4	6, 12 and 24	Infection
Mener et al. [34]	2020	Georgia	Retrospective	259	NR					24	Infection
Reuss and Cole [42]	2007	US	Retrospective	81	14	19	9	34	5	8	Deep infection Non-union
Singh et al. [31]	2012	UK	Retrospective	67	0	0	26	39	2	6	Deep infection Non-union
Spencer et al. [43]	2004	UK	Prospective	41	0	5	14	11	0	6	Deep infection Non-union
Sungaran et al. [35]	2007	Australia	Retrospective	161	28	35	95			6 and 12	Infection
Tripuraneni et al. [36]	2008	US	Retrospective	215	62	98	26	25	4	6, 12 and 24	Infection
			Total	10,032	321	531	819				

NR = Not Reported; UK = United Kingdom; US = United States. Fractures and Gustilo-Anderson classification are reported as frequency. Time threshold portrays the time threshold used (in hours) to differentiate between early and late debridement.

**Table 2:** Summary of findings table for primary outcomes (infection and non-union) with relative and anticipated absolute effects, GRADE quality of evidence assessment and evidence interpretation.

Outcome	No of Participants (Studies)	Relative Effect (95% CI)	*p*-Value	Anticipated Absolute Effects (95% CI)			Certainty (GRADE)	Interpretation
				Early Debridement	Late Debridement	Difference		
Infection	2193 (17)	OR 0.87 (0.68 to 1.11)	0.23	14.0%	12.4% (10 to 15.3)	1.6% fewer (4 fewer to 1.3 more)	⨁◯◯◯ VERY LOW ^a,b,c^	The evidence suggests that late debridement results in little to no difference in infection.
Non-union	817 (7)	OR 0.70 (0.42 to 1.15)	0.13	16.8%	12.4% (7.8 to 18.8)	4.4% fewer (9 fewer to 2 more)	⨁◯◯◯ VERY LOW ^a,c,d^	The evidence suggests that late debridement results in little to no difference in non-union.

The risk in the intervention group (and its 95% confidence interval) is based on the assumed risk in the comparison group and the relative effect of the intervention (and its 95% CI). CI: Confidence interval; OR: Odds ratio. Low certainty: Our confidence in the effect estimate is limited: The true effect may be substantially different from the estimate of the effect. Explanations: ^a^ Serious risk of bias assessment as most studies were judged to carry serious bias (n = 13), while the rest 7 stuies moderate. ^b^ Serious indirectness as most studies did not define how debridement was carried out. Method of assessing infetion varied and was not standardised. ^c^ Serious imprecision as although size sample is large, the 95% CI overlaps with no effect (OR = 0). ^d^ Serious indirectness as most studies did not define how debridement was carried out. Method of confirming non-union varied and was not standardised.

## Data Availability

The data presented in this study are available upon a reasonable request from the corresponding author.

## Supplementary Materials

The following are available online at https://www.mdpi.com/article/10.3390/diagnostics11061017/s1, Figure S1: Risk of bias assessment results using the ROBINS-I tool for non-randomised controlled trials; Table S1: Keyword Strategy used for Medline (via Ovid) on 13 May 2020; Table S2: Keyword Strategy used for Embase on 13 May 2020; Table S3: Keyword Strategy used for PubMed on 13 May 2020; Table S4: Keyword Strategy used for Scopus on 13 May 2020; Table S5: Keyword Strategy used for Cochrane Central on 13 May 2020; Table S6: Keyword Strategy used for Web of Science Core Collection on 13 May 2020.
