# Supplementary material for: The Effect of Delayed Surgical Debridement in the Management of Open Tibial Fractures: A Systematic Review and Meta-Analysis
Source: Diagnostics (Basel). 2021 Jun 2;11(6):1017. doi: 10.3390/diagnostics11061017 (PMC8228778; doi:10.3390/diagnostics11061017)
Supplement: Supplementary file 1 [file diagnostics-11-01017-s001.zip › Supplementary File 1.pdf]

MEDLINE (via Ovid) – 13th May 2020

#	Search term	Results
1	exp Fractures, Open/ (MeSH)	5374
2	Open fracture?	4971
3	Open ADJ3 fracture?	10956
4	#1 OR #2 OR #3	10956
5	Tibia?	98778
6	exp Tibia/ (MeSH)	34683
7	#5 OR #6	98778
8	#4 AND #7	3467
9	Open tibia? Fracture?	734
10	#8 OR #9	3467
11	exp Debridement/ (MeSH)	16113
12	Debridement	32421
13	#11 OR #12	32421
**14**	**#10 AND #13**	**546**

EMBASE – 13th May 2020

#	Search term	Results
1	‘Open fracture’/exp	6997
2	‘Open fracture*’	10012
3	Open NEAR/3 fracture*	13180
4	#1 OR #2 OR #3	13180
5	Tibia*	135309
6	‘Tibia’/exp	51251
7	#5 OR #6	135518
8	#4 AND #7	3927
9	'surgical debridement'/exp	652
10	Debridement	52506
11	#9 OR #10	52506
**12**	**#8 AND #11**	**770**

PubMed – 13th May 2020

#	Search term	Results
1	"Fractures, Open"[Mesh]	5374
2	“Open fracture*”	2257
3	Open NEAR/3 fracture	100
4	#1 OR #2 OR #3	7023
5	Tibia*	107928
6	"Tibia"[Mesh]	34685
7	#5 OR #6	107928
8	#4 AND #7	2428
9	"Debridement"[Mesh]	16116
10	Debridement	32330
11	#9 OR #10	32330
**12**	**#8 AND #11**	**438**

SCOPUS – 13th May 2020

#	Search term	Results
1	"Open fracture*"	18582
2	Open W/3 fracture*	35061
3	#1 OR #2	35061
4	Tibia*	253696
5	#3 AND #4	13619
6	Debridement	75647
**7**	**#5 AND #6 (LIMIT TO TITLE/ABSTRACT/KEYWORDS)**	**894**

COCHRANE CENTRAL – 13th May 2020

#	Search term	Results
1	‘Open fracture’/exp	120
2	‘Open fracture*’	3533
3	Open NEAR/3 fracture*	768
4	#1 OR #2 OR #3	3533
5	Tibia*	5743
6	‘Tibia’/exp	576
7	#5 OR #6	5743
8	#4 AND #7	451
9	'debridement'/exp	610
10	Debridement	2981
11	#9 OR #10	2981
**12**	**#8 AND #11 (trials only)**	**35**

Web of Science Core Collection – 13th May 2020

#	Search term	Results
1	TS=("Open fracture$")	3929
2	TS=(Open NEAR/3 fracture$)	9194
3	#1 OR #2	9194
4	TS=(Tibia*)	80297
5	#3 AND #4	2527
6	TS=("Open tibia* Fracture$")	1057
7	#5 OR #6	2527
8	TS=(Debridement)	20907
**9**	**#7 AND #8**	**416**
