# Supplementary figures and images for: The Effect of Delayed Surgical Debridement in the Management of Open Tibial Fractures: A Systematic Review and Meta-Analysis
Source: Diagnostics (Basel). 2021 Jun 2;11(6):1017. doi: 10.3390/diagnostics11061017 (PMC8228778; doi:10.3390/diagnostics11061017)

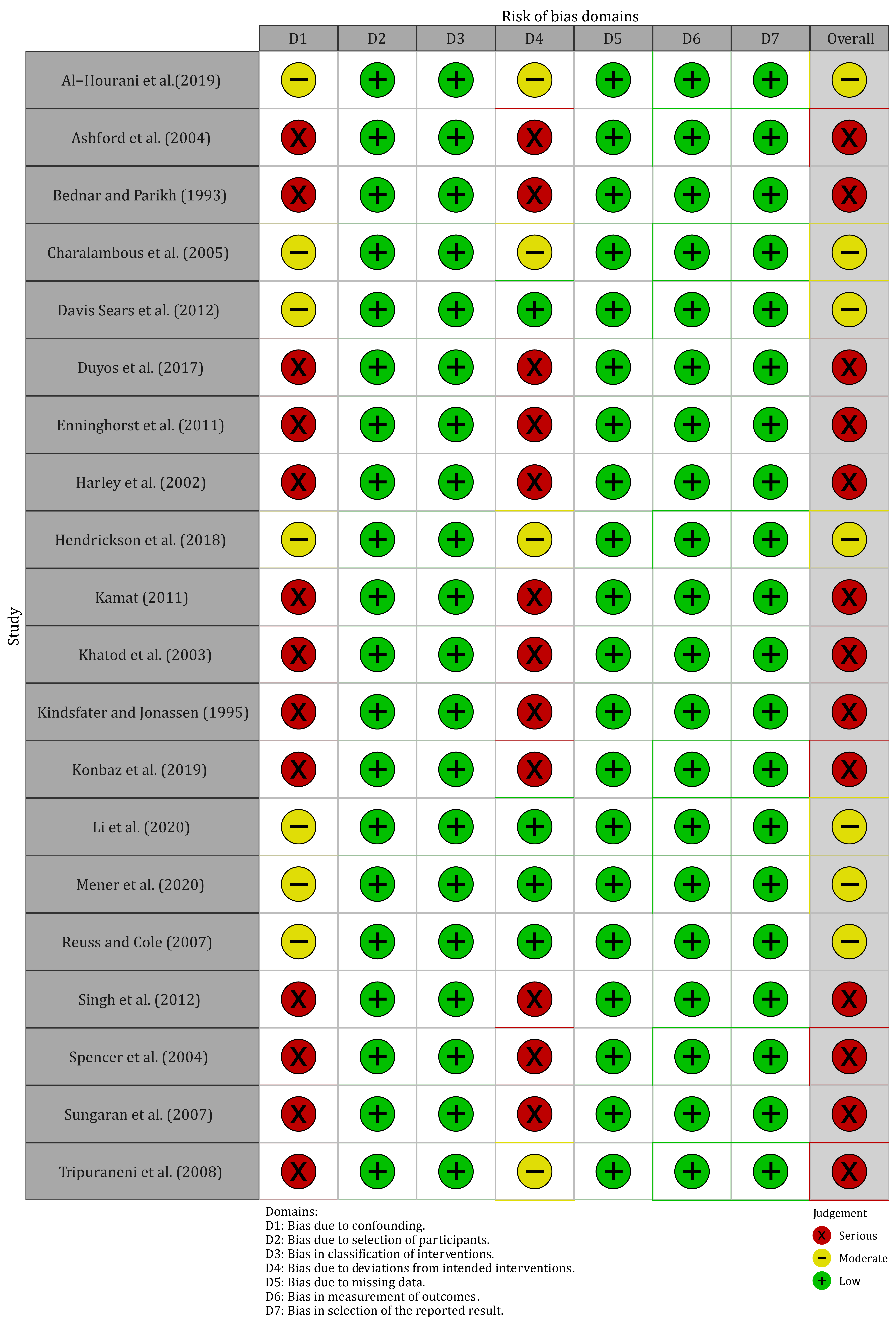

Supplement: Supplementary file 1 [file diagnostics-11-01017-s001.zip › Supplementary File 2.png]
